# Maternal obesity is associated with a reduction in placental taurine transporter activity

**DOI:** 10.1038/ijo.2014.212

**Published:** 2015-01-20

**Authors:** A M Ditchfield, M Desforges, T A Mills, J D Glazier, M Wareing, K Mynett, C P Sibley, S L Greenwood

**Affiliations:** 1 Maternal and Fetal Health Research Centre, Institute of Human Development, University of Manchester, Manchester, UK; 2 Maternal and Fetal Health Research Centre, St. Mary's Hospital, Central Manchester University Hospitals NHS Foundation Trust, Manchester Academic Health Science Centre, Manchester, UK

## Abstract

**Background/Objectives:**

Maternal obesity increases the risk of poor pregnancy outcome including stillbirth, pre-eclampsia, fetal growth restriction and fetal overgrowth. These pregnancy complications are associated with dysfunctional syncytiotrophoblast, the transporting epithelium of the human placenta. Taurine, a β-amino acid with antioxidant and cytoprotective properties, has a role in syncytiotrophoblast development and function and is required for fetal growth and organ development. Taurine is conditionally essential in pregnancy and fetal tissues depend on uptake of taurine from maternal blood. We tested the hypothesis that taurine uptake into placental syncytiotrophoblast by the taurine transporter protein (TauT) is lower in obese women (body mass index (BMI)⩾30 kg m^−^^2^) than in women of ideal weight (BMI 18.5–24.9 kg m^−^^2^) and explored potential regulatory factors.

**Subjects/Methods:**

Placentas were collected from term (37–42-week gestation), uncomplicated, singleton pregnancies from women with BMI 19–49 kg m^−^^2^. TauT activity was measured as the Na^+^-dependent uptake of ^3^H-taurine into placental villous fragments. TauT expression in membrane-enriched placental samples was investigated by western blot. *In vitro* studies using placental villous explants examined whether leptin or IL-6, adipokines/cytokines that are elevated in maternal obesity, regulates TauT activity.

**Results:**

Placental TauT activity was significantly lower in obese women (BMI⩾30) than women of ideal weight (*P*<0.03) and inversely related to maternal BMI (19–49 kg m^−^^2^; *P*<0.05; *n*=61). There was no difference in TauT expression between placentas of ideal weight and obese class III (BMI⩾40) subjects. Long-term exposure (48 h) of placental villous explants to leptin or IL-6 did not affect TauT activity.

**Conclusions:**

Placental TauT activity at term is negatively related to maternal BMI. We propose that the reduction in placental TauT activity in maternal obesity could lower syncytiotrophoblast taurine concentration, compromise placental development and function, and reduce the driving force for taurine efflux to the fetus, thereby increasing the risk of poor pregnancy outcome.

## Introduction

Around 1 in 5 women in the UK are obese at the start of pregnancy (body mass index (BMI): kg m^−^^2^) >30)^1^ and 1 in 1000 expectant mothers has a BMI of ⩾50 (morbidly obese) at delivery.^2^ Maternal obesity increases the likelihood of serious pregnancy complications both for mother and infant. As the prevalence of obesity is increasing in women of reproductive age, it is probable that associated pregnancy complications will increase in parallel. Obesity during pregnancy is associated with an elevated risk of developing pre-eclampsia, a leading cause of maternal and fetal death and fetal growth restriction (FGR).^3, 4^ Compared with women of normal BMI (18.5–24.9), the risk of pre-eclampsia is tripled at BMI 30 (obese class I) and increased fourfold at BMI 40 (obese class III).^5^ However, maternal obesity is an independent risk factor for stillbirth, often associated with growth restriction, and for excessive fetal growth (macrosomia).^6, 7^ As disorders of fetal growth predispose the individual to obesity, metabolic syndrome and cardiovascular disease in adulthood, maternal obesity has a major negative effect on the health of the next generation.^8, 9^

Successful pregnancy depends on appropriate development and function of the placenta to ensure adequate delivery of oxygen and nutrients from mother to fetus.^10^ Pre-eclampsia and disorders of fetal growth (FGR, macrosomia) are associated with placental dysfunction,^11^ and maternal obesity is likely to increase the risk of these pregnancy complications through effects on the placenta. However, mechanisms underlying the disparate pregnancy complications associated with maternal obesity remain poorly researched.^12^ Maternal-fetal nutrient transfer is mediated by syncytiotrophoblast, the transporting epithelium of the human placenta, which has a microvillous plasma membrane in contact with maternal blood and a basal plasma membrane adjacent to the fetal capillaries.^13^ Over the course of pregnancy, syncytiotrophoblast undergoes renewal by proliferation, differentiation/fusion and incorporation of underlying cytotrophoblast cells. In normal pregnancy, syncytiotrophoblast is maintained in a steady state where the relative numbers of cytotrophoblast and syncytiotrophoblast nuclei remain constant. Abnormalities in proliferation, fusion and apoptosis dysregulate syncytiotrophoblast and potentially limit the transfer of nutrients to the fetus.^14^ Indeed, abnormal syncytiotrophoblast renewal is evident in women with reduced fetal movements, a risk factor for stillbirth,^15^ pre-eclampsia and FGR,^16^ as well as in women with raised BMI.^17^ Accordingly, dysregulated syncytiotrophoblast renewal could link maternal obesity and development of pregnancy complications.

There is evidence that the altered maternal environment in women with a raised BMI could have adverse consequences for placental development and function. Adipose tissue is a major source of adipokines/inflammatory cytokines and, in common with obesity in the non-pregnant individual, obesity in pregnancy is characterised by heightened inflammation and altered endocrine secretion. For example, maternal circulating levels of leptin and IL-6 are increased, whereas adiponectin levels are reduced,^18, 19, 20^ disrupting the normal adaptation in maternal endocrine milieu associated with pregnancy.^21^ Maternal obesity is also associated with heightened levels of placental oxidative^22^ and nitrative^23^ stress, conditions that dysregulate syncytiotrophoblast renewal *in vitro*.^24, 25^ Finally, plasma amino-acid concentrations in pregnant obese women may be different to women of ideal weight,^26^ altering the availability of nutrients for maternal–fetal transfer across the placenta.

The β-amino-acid taurine (2-aminoethanesulfonic acid), although not a constituent of proteins,^27^ is essential for fetal growth and organogenesis.^28, 27^ The enzyme required for taurine synthesis is absent in human fetal tissues^29^ and their requirement for taurine is met by uptake from maternal blood via the taurine amino-acid transporter (TauT) on the syncytiotrophoblast microvillous plasma membrane.^30^ Taurine has many important physiological functions that include facilitating cell volume regulation, critical for proliferation and apoptosis, and cytoprotection from ischemia-reperfusion injury, inflammation, hypoxia and oxygen-free radicals.^31, 32^ We showed that the cytotrophoblast differentiation *in vitro* was compromised in taurine-deficient cells and that susceptibility of these cells to an inflammatory stimulus was increased, as evidenced by elevated levels of apoptosis.^33^ Therefore, taurine is likely to have a critical role not only in fetal development but also in the development and function of the placenta by facilitating syncytiotrophoblast renewal and cell survival.

The activities of several placental amino-acid transporters, including TauT, have been extensively studied in relation to FGR^34, 30, 35^ but, with the exception of the system A amino-acid transporter,^36^ little attention has been paid to their activities/expression in relation to maternal BMI. Stillbirth, FGR and pre-eclampsia associated with obesity may be a consequence of limited delivery of essential amino acids, including taurine, to the placenta and fetus. We tested the hypothesis that the activity/expression of TauT is lower in placentas of obese (BMI⩾30) compared with ideal weight women (BMI 18.5–24.9). In addition, we investigated whether long-term exposure to leptin and IL-6, which are elevated in maternal obesity, modulates placental TauT activity *in vitro.*

## Materials and methods

### Materials

Unless stated otherwise, chemicals and reagents were purchased from Sigma Aldrich, Gillingham, UK.

### Study participants and tissue collection

Women were recruited from the Central Delivery Unit at St. Mary’s Hospital, Manchester and gave written informed consent as approved by the Local Research Ethics Committee. Participants had BMI recorded at their first antenatal visit (<12 weeks of pregnancy). Women with pre-existing or pregnancy-related complications, including an abnormal glucose tolerance test, were excluded. Placentas were collected within 15–20 min of vaginal or Caesarean delivery from full-term (37–42-week gestation) uncomplicated, singleton pregnancies delivering between the 10th and 90th individualized birth weight centile (birth weight corrected for parity, gestation, maternal ethnicity, height and weight^37^). Fetal membranes were trimmed to the placental margins, the umbilical cord was removed at the point of insertion and the placenta was weighed.

### Measurement of placental TauT activity

TauT activity was measured in placental villous fragments as described previously.^38^ In brief, randomly sampled villous tissue fragments were maintained in 1:1 DMEM/Tyrode’s solution (135 mm NaCl, 5 mm KCl, 1.8 mm CaCl_2_, 1.0 mm MgCl_2_(6 H_2_O), 10 mm HEPES and 5.6 mm D-glucose, pH 7.4) at 37 °C for 30 min for temperature equilibration. Following a 2-min wash, fragments were transferred into Na^+^-containing or Na^+^-free Tyrode’s buffer (latter to measure Na^+^-independent uptake; NaCl replaced by 135 mm choline chloride) containing 0.037 MBq ml^−1^ (50 pmol ml^−1^) ^3^H-taurine (Amersham Biosciences, Little Chalfont, UK). Uptake of ^3^H-taurine was measured for 30, 60, 90 and 120 min and then stopped by washing the fragments in ice-cold Na^+^-containing or Na^+^-free Tyrode’s buffer as appropriate. The tissue was lysed in distilled water (18 h; room temperature) to release accumulated isotope and then denatured in 0.3 m NaOH overnight (37 °C) for determination of fragment protein content (Bradford method^39^). ScintiSafe 2 High Performance Liquid scintillation cocktail (Fisher Scientific, Loughborough, UK) was added to the water lysate and radioactivity measured using a Tri-Carb 2100 TR scintillation counter (Packard Bioscience). As TauT is a Na^+^-dependent transporter, TauT activity was calculated as the difference between ^3^H-taurine uptake in control and Na^+^-free conditions (nmol mg^−1^ protein).

### Western blotting

Placental TauT expression was compared in ideal weight and obese class III subjects (BMI⩾40) from placentas in which TauT activity was determined. Membrane-enriched samples were prepared and western blot analysis of TauT protein expression was carried out as described previously^40, 41^ using a rabbit anti-TauT affinity purified polyclonal antibody (Millipore, Nottingham, UK; 1:400 dilution; 2.5 mg ml^−1^). Samples were mixed with reducing buffer (10% 1 m Tris-HCl, 4% SDS, 20% glycerol, 0.040% bromophenol blue, 2% beta-mercaptoethanol) in a 2:1 ratio and heated at 95 °C for 5 min. Nitrocellulose membranes were stripped and re-probed with a rabbit polyclonal anti-β-actin antibody AC-15 clone (1:1500 dilution) to validate protein loading and to confirm protein integrity of the samples. Primary and horseradish peroxidase-conjugated secondary antibody (1:1000 dilution, Dako Ltd., Ely, UK) incubations were performed for 1 h at room temperature. Positive signals were detected using enhanced chemiluminescence and the relative densities of the immunoreactive species were evaluated using Image J image processing software version 1.44 (National Institutes of Health, http://www.imagej.nih.gov). To account for any variability in sample loading, TauT signal intensity in each sample was normalized to the corresponding β-actin signal intensity.

### Culture of human placental villous explants

Villous tissue was sampled from placentas of ideal weight women having normal pregnancy and maintained in culture according to Siman *et al.*^42^ Explants of villous tissue (~2.5 mm^3^) were washed in phosphate-buffered saline to remove maternal blood and transferred to Netwell permeable supports (70 μm mesh; Corning Lifesciences, Amsterdam, The Netherlands) in 12-well culture dishes. Explants 3 × per Netwell were maintained at the liquid/air interface in 1.5 ml culture medium (100 ml l^−1^ 10xCMRL 1066, 2.2 g l^−1^ NaHCO_3_, 100 μg ml^−1^ streptomycin sulphate, 100 IU ml^−1^ penicillin G, 0.1 μg ml^−1^ hydrocortisone, 1 μg ml^−1^ insulin, 0.1 μg ml^−1^ retinol acetate, 100 mg l^−1^ L-glutamine, 5% fetal bovine serum, pH 7.2) at 37 °C (5% CO_2_/air) for 7 days. Medium was collected daily to measure human chorionic gonadotrophin (hCG) secretion, used as endocrine marker of syncytiotrophoblast regeneration.^42^ On day 7 of culture, TauT activity was determined as the Na^+^-dependent ^3^H-taurine uptake into the explants (over 90 min) as described above.

CRML 1066 medium does not contain taurine and, as TauT activity in placental cells is adaptively upregulated by low extracellular taurine,^43^ syncytiotrophoblast TauT activity in explants could be upregulated in CRML 1066 culture medium (containing 10–25 μm taurine contributed by serum). To avoid adaptive regulation of TauT, 100 μm taurine was added to CRML 1066 to approximate taurine in maternal blood (14–160 μm^44^). To assess whether TauT activity in explants cultured with 100 μm taurine was similar to that *in situ*, TauT activity at day 7 of culture in medium containing 100 μm taurine was compared with activity in fresh tissue fragments prepared from the same placenta.

### *In vitro* studies on TauT activity: effects of leptin and IL-6

Long-term effects (48 h) of leptin and IL-6 on TauT activity were measured in villous explants (medium contained 100 μm taurine). Leptin (1 mg) was diluted in 0.3 ml 7.5 mm NaOH and 0.5 ml 15 mm HCl and stored at −20 °C. IL-6 (10 μg) was diluted in 200 μl phosphate-buffered saline with 1% bovine serum albumin and stored at −20 °C. Stocks were diluted in CMRL culture medium to give working concentrations of 50 or 500 ng ml^−1^ leptin and 2 or 20 pg ml^−1^ IL-6 to span the range of plasma concentrations in maternal obesity.^19, 20^ Culture medium alone served as a control. Leptin or IL-6 was added on days 5 and 6 and TauT activity was measured on day 7 of culture.

### Human chorionic gonadotrophin analysis

The concentration of hCG in culture medium was measured by enzyme-linked immunosorbent assay (DRG Diagnostics, Marburg, Germany) according to the manufacturer’s instructions. Explant protein content was determined on day 7 (Bradford method^39^) and hCG secretion expressed as mIU per mg explant protein per hour of culture.

### Statistical analysis

The Kolmogorov–Smirnov test was used to determine whether data were normally distributed. The time course of Na^+^-dependent ^3^H-taurine uptake (TauT activity) was compared between the BMI groups using least squares linear regression. Differences in TauT activity at 120 min, TauT protein expression, and demographic data were evaluated using a Mann–Whitney *U* or Kruskal–Wallis test. The Wilcoxon signed rank test was used to assess whether leptin or IL-6 significantly influenced TauT activity. Data analyses were performed using Graph Pad Prism Software (GraphPad Inc., La Jolla, CA, USA); *P*<0.05 was considered statistically significant.

## Results

### Study participants

Participants were divided into their respective BMI categories, according to WHO classifications, and their demographic details are presented in Table 1. Maternal age and parity did not differ between the BMI groups (Kruskal–Wallis test: *P*>0.05). There was a mix of ethnic groups in each BMI category but ~60% were white British in the ideal weight, overweight and obese groups. Five out of twenty-seven obese women were current smokers and most women delivered by pre-labor Caesarean section. Table 2 shows the characteristics of the infants of the study participants. Birth weight, placental weight, fetal/placental weight ratio, individualized birth weight centile and gestational age did not differ between the BMI groups (Kruskal–Wallis test: *P*>0.05).

**Figure 1:**
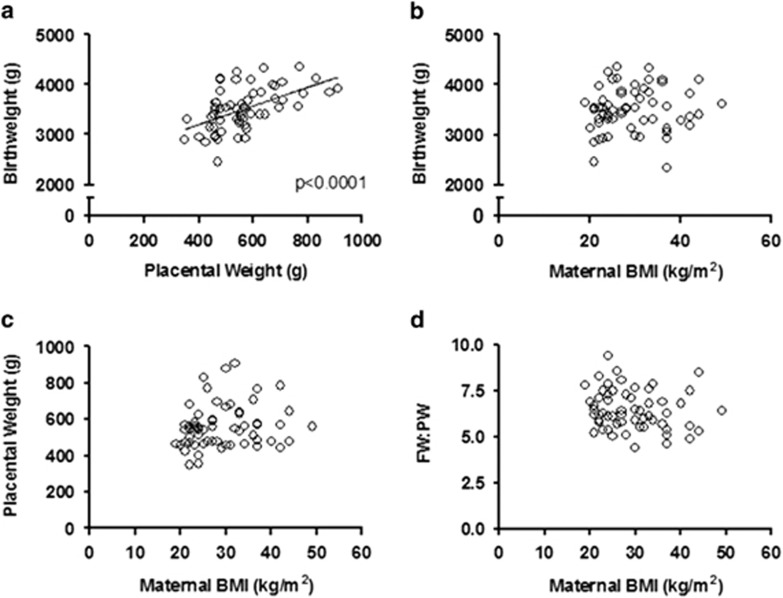
(**a**) Birth weight was positively related to placental weight (least squares linear regression: *r*^2^=0.29, *P*<0.0001; *n*=61). Maternal BMI was unrelated to (**b**) birth weight, (**c**) placental weight and (**d**) fetal/placental weight ratio (FW:PW; least squares linear regression analysis; *P*>0.05; *n*=61).

In the total study group (*n*=61) there was a significant positive relationship between placental weight and birth weight (Figure 1a) and between placental weight and individualized birth weight centile (least squares linear regression: *r*^2^=0.21, *P*<0.0002; data not shown). Maternal BMI was unrelated to birth weight (between the 10th and 90th centile), placental weight and fetal/placental weight ratio (Figure 1b–d respectively).

### TauT activity in relation to BMI

**Figure 2:**
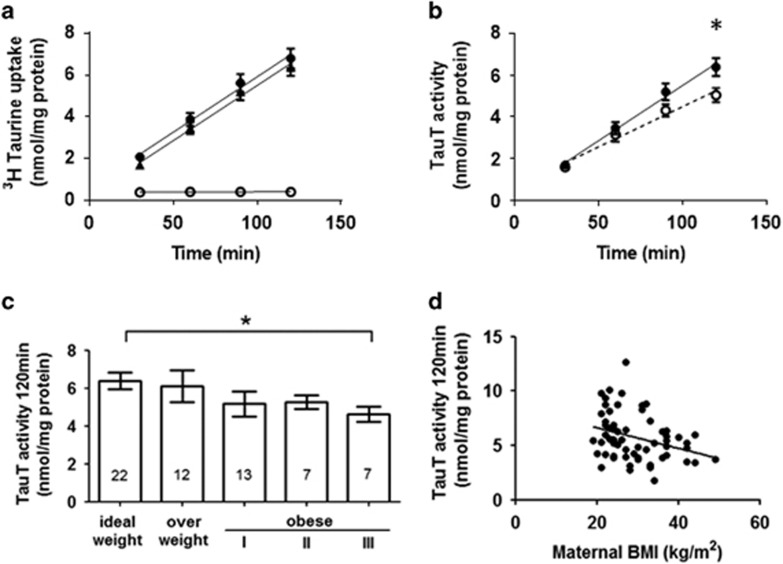
(**a**) Uptake of ^3^H-taurine into villous fragments in the presence (●) and absence (○) of Na^+^ over 30–120 min in the placentas of ideal weight women. The Na^+^-dependent component of uptake (▴) indicates ^3^H-taurine uptake owing to TauT activity. There was a linear relationship between TauT activity and time over 30–120 min (least squares linear regression: *r*^2^=0.56, *P*<0.0001). (**b**) Placental TauT activity was significantly lower in obese women (BMI⩾30 kg m^−2^; *n*=27 (○)) compared with women of ideal weight (BMI 18.5–24.9 kg m^−2^; *n*=22 (●)) (mean±s.e.; *P<0.03 least squares linear regression). (**c**) Placental TauT activity (120 min) according to the BMI category (mean±s.e.; *P<0.05; Kruskall–Wallis with Dunn’s multiple comparison *post hoc* test). (**d**) Placental TauT activity (120 min) was inversely related to maternal BMI (least squares linear regression: *r*^2^=0.098; *P*<0.014 *n*=61).

The uptake of ^3^H-taurine into villous fragments is shown in Figure 2a using placentas from women of ideal weight as an example. TauT activity, the Na^+^-dependent component of ^3^H-taurine uptake, was linear up to 120 min for all categories of BMI indicating that activity was measured at initial rate (data not shown). TauT activity (30–120 min) was significantly lower in placentas of obese (BMI⩾30) as compared with ideal weight women (Figure 2b; 21% lower at 120 min). As TauT activity was linear up to 120 min, this time point was selected for subsequent analyses. To explore the relationship between BMI category and TauT activity, subjects with a BMI ⩾30 were divided into their obesity subgroups (Figure 2c). There was no difference in TauT activity in women who were overweight compared with women of ideal weight. There was a reduction in TauT activity in obesity and this was significantly lower in placentas of obese class III women (BMI⩾40) compared with women of ideal weight. When TauT activity was related to maternal BMI as a continuous variable, a significant negative relationship was observed over the BMI range 19–49 (Figure 2d).

The effect of mode of delivery on TauT activity was determined as the majority of obese women delivered by Caesarean section. Twelve out of fourteen deliveries were by Caesarean section in the obese class II and III groups precluding statistical comparison, but in the ideal weight, overweight and obese class I groups, TauT activity was unaffected by mode of delivery (*P*>0.05; data not shown). Furthermore, the significant negative relationship between placental TauT activity and maternal BMI remained evident when women who had Caesarean section delivery were analyzed (*P*<0.05; data not shown). TauT activity was unaffected by maternal smoking and parity (*P*>0.05; data not shown).

### TauT protein expression

**Figure 3:**
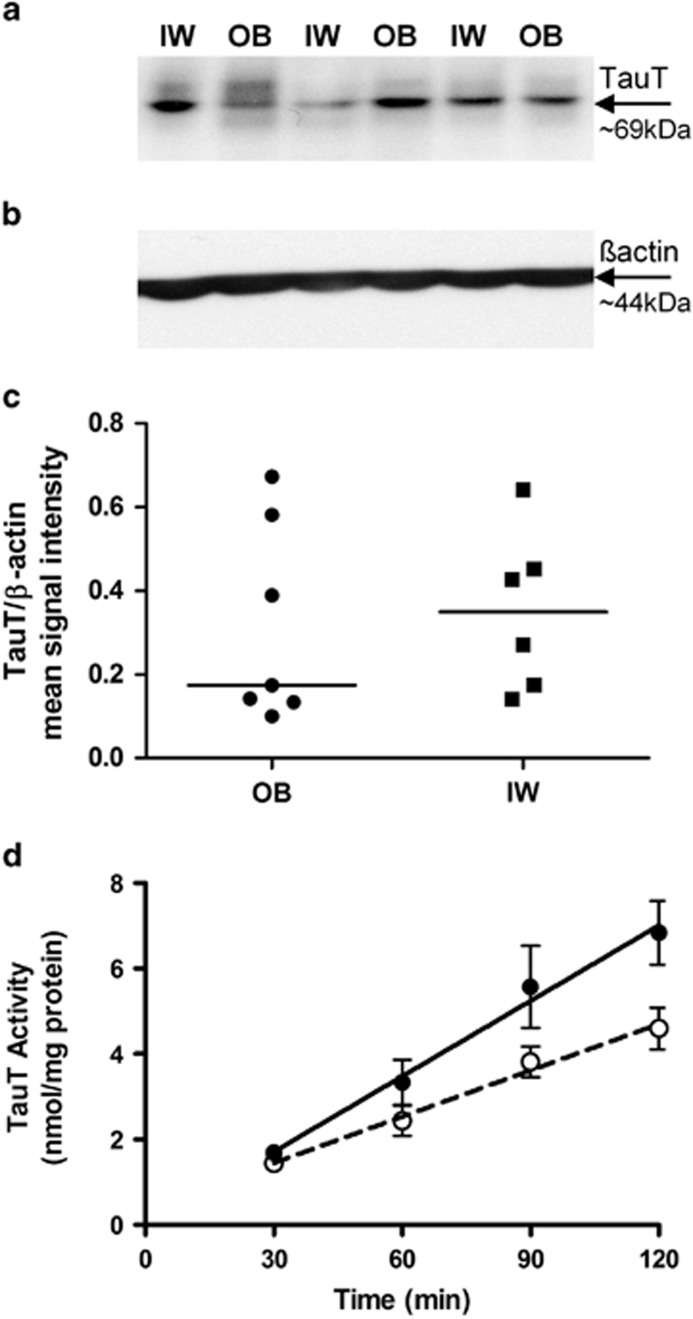
Representative western blots of placental membrane-enriched fractions from three women of ideal weight (IW) and three women who were obese class III (BMI⩾40 kg m^−2^: obese, OB) subjects probed for TauT and β-actin. Protein loading was 50 μg per lane. (**a**) After probing for TauT, an immunoreactive species was detected at ~69 kDa in all samples. (**b**) After re-probing membranes for β-actin, an immunoreactive species was detected at ~44 kDa. (**c**) Scatter plots display densitometric analysis of TauT signal intensity after normalizing to β-actin (line at median). There was no significant difference in TauT protein expression between IW (*n*=6) and OB (*n*=7) groups (Mann–Whitney *U-*test: *P*>0.05). (**d**) Placental TauT activity, in the same placentas in which TauT expression was determined, was significantly lower in class III obese women (*n*=7; (○)) than women of IW (*n*=6; (○)) (mean±s.e.; *P<0.05, least squares linear regression).

Figure 3a shows representative western blots of TauT expression in membrane-enriched placental samples from ideal weight (*n*=6) and obese class III (*n*=7) subjects in which TauT activity was significantly reduced (Figure 3d). After probing with anti-TauT antibody, an immunoreactive signal for TauT was detected at ~69 kDa (Figure 3a), corresponding to the molecular mass of the protein encoded by the predominant TauT transcript in the placenta.^45^ To confirm protein integrity and correct for protein loading, membranes were probed for β-actin and an immunoreactive species was detected at ~44 kDa (Figure 3b). Densitometry of TauT expression normalized to β-actin revealed that there was no difference in TauT expression in placentas of ideal weight and obese class III subjects (Figure 3c).

### *In vitro* studies on TauT activity: effect of taurine concentration and adipokines

**Figure 4:**
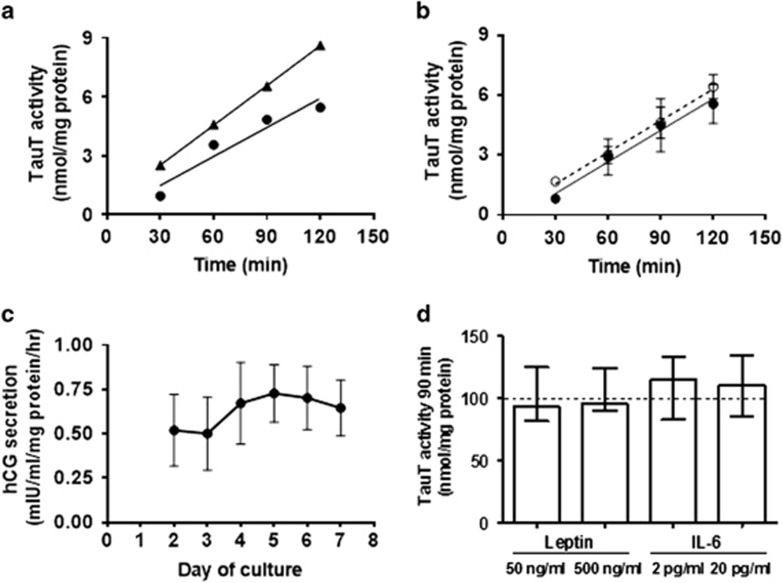
(**a**) TauT activity at day 7 of culture is higher in placental villous explants maintined in medium with no added taurine (▴) compared with that with 100 μm added taurine (●). (**a**) Representative culture (least squares linear regression). (**b**) TauT activity in explants at day 7 of culture in medium with 100 μm added taurine (●) was not significantly different from activity determined in freshly isolated tissue fragments from the same placenta (**○**) (mean±s.e.; *n*=3 placentas; least squares linear regression). (**c**) Time course of hCG secretion by untreated placental villous explants over 7 days of culture (mean±s.e.; *n*=5) indicated endocrine viability. (**d**) TauT activity (90 min) in explants at day 7 of culture, expressed as a percentage of the controls (100% dotted line), was unaffected by 48 h exposure to either leptin or IL-6 (Wilcoxon signed rank test vs control (100%): median and interquartile range; *n*=5).

Figure 4a shows an example of TauT activity in explants cultured in standard medium (10–25 μm taurine contributed by serum) and medium with 100 μm taurine to simulate maternal plasma taurine concentration. As previously reported,^46, 43^ TauT activity was upregulated following culture in low-taurine conditions. Figure 3b shows that there was no difference in TauT activity after 7 days of explant culture in medium containing 100 μm taurine and activity in freshly isolated villi from the same placenta. This confirms that the addition of 100 μm taurine in culture restores TauT activity to normal. Subsequent experiments to assess effects of leptin and IL-6 were carried out using the explants cultured in medium with 100 μm taurine.

Over the first 2 days of explant culture, syncytiotropoblast sheds and thereafter regenerates to form new syncytiotrophoblast that is morphologically indistinguishable from normal by day 7. This process is accompanied by temporal changes in hCG secretion that is low on day 2 of culture and increases with regeneration of syncytiotrophoblast on days 5–7.^47, 42^ Figure 4c shows that hCG secretion increased over days 3–5 of culture, as previously described,^47, 42^ indicating syncytiotrophoblast regeneration and explant endocrine viability. Treatment with leptin and IL-6 on days 5/6 of culture did not alter hCG secretion (data not shown).

Figure 4d shows that neither leptin nor IL-6 (48 h) altered TauT activity measured at day 7 of culture when expressed as a percentage of the corresponding untreated control (100%).

## Discussion

This study demonstrates that the placental TauT activity is negatively related to maternal BMI, with the greatest reduction in activity in women with a BMI ⩾40 compared with their ideal weight counterparts. Our findings indicate that taurine uptake by the placenta and delivery to the fetus may be compromised in obese pregnant women. Taurine facilitates the maintenance of syncytiotrophoblast and the reduction in TauT activity could contribute to placental dysfunction in maternal obesity and increase susceptibility to pregnancy complications.

A recent study of 55 105 women demonstrated that the placental weight and birth weight increased, and fetal:placental weight ratio (indicating placental efficiency) decreased incrementally with increasing maternal BMI.^48^ In the current small cohort of women delivering appropriately grown babies, placental weight, birth weight and fetal:placental weight ratio were unrelated to BMI. Therefore, reduced placental TauT activity (per mg placenta) in obesity was not a compensation for increased placental size. Assuming taurine uptake reflects transport across the placenta, a reduction in TauT activity in the absence of increased placental mass would reduce taurine delivery to the fetus. Fetal plasma taurine concentration was not measured in the present study and it remains to be determined whether babies born to obese women have lower plasma taurine concentration than those born to women of ideal weight. However, reduced placental TauT activity is evident in FGR^30, 35^ and FGR fetuses have lower plasma taurine concentration than fetuses that are normally grown.^49^ In the current study, downregulation of TauT activity was observed in placentas of babies that were an appropriate size for gestational age at birth. However, as animal studies show that taurine is essential for fetal organogenesis and subsequent offspring health,^28, 27^ taurine depletion in fetuses of obese mothers could negatively impact on organ development and thus the wellbeing of the individual in later life.

In addition to compromising maternal–fetal taurine transfer, a reduction in intracellular taurine, consequent on reduced TauT activity in maternal obesity, could impair maintenance of syncytiotrophoblast. We have previously investigated the effect of inhibiting TauT activity on formation of syncytiotrophoblast by cytotrophoblast cells in primary culture. Small interfering RNA-mediated knockdown of TauT reduced intracellular taurine and inhibited the fusion/differentiation of cytotrophoblast cells to form syncytia.^33^ Furthermore, susceptibility of cells to apoptosis in response to tumor necrosis factor-α was significantly enhanced following TauT knockdown,^33^ demonstrating that reduced intracellular taurine increases the vulnerability of syncytiotrophoblast to damage by an inflammatory cytokine that is elevated in maternal obesity.^18^ In a more recent study, we investigated the effects of inhibiting TauT activity on the regeneration of syncytiotrophoblast in placental villous explants over 7 days of culture. Competitive inhibition of taurine uptake reduced intracellular taurine and this was accompanied by significantly reduced syncytiotrophoblast regeneration.^50^ We hypothesize that reduced placental taurine accumulation in maternal obesity could contribute to abnormal syncytiotrophoblast renewal,^17^ with detrimental consequences for placental function and fetal development. However, it remains to be demonstrated that the reduction in placental taurine uptake in obesity lowers syncytiotrophoblast taurine concentration.

The reduction in placental TauT activity in obesity was not associated with lower protein expression. TauT expression was similar in women with a BMI ⩾40 and women of ideal weight, despite a reduction in TauT activity of 33% in the same placentas. As maternal obesity is associated with an elevated plasma concentrations of leptin and IL-6,^19, 20^ we explored the possibility that TauT activity was downregulated by long-term application of these using the placental explant model.^47, 42^ In common with a previous study, where short-term exposure (1 h) to leptin and IL-6 did not alter TauT activity in villous fragments,^35^ 48-h exposure to either leptin or IL-6 did not affect TauT activity in explants. Thus, it is unlikely that reduced placental TauT activity in women with raised BMI is mediated by an elevated plasma leptin or IL-6 concentration.

In many cell types including trophoblast,^43^ TauT activity adaptively downregulates in response to elevated extracellular taurine concentration. In the current study, we demonstrated that this adaptive regulation is evident in intact syncytiotrophoblast (Figure 4a). In preliminary studies, we also found that maternal plasma taurine concentration at term was significantly higher in obese than in ideal weight women (38±2.6 μm; *n*=13 vs 29.6±2.3 μm; *n*=11, respectively^51^), raising the possibility that reduced placental TauT activity in obesity may be an adaptive response to elevated maternal plasma taurine concentration. It remains to be determined whether the magnitude of increase in plasma taurine observed in obese women is sufficient to induce an adaptive response.

A reduction in TauT activity but not expression suggests post-translational downregulation of TauT in maternal obesity. TauT activity in non-placental tissues is inhibited by phosphorylation of serine 322 on the TauT protein that can be induced by protein kinase C (PKC).^52^ Activation of PKC inhibits TauT activity in placental villous tissue^41, 53, 35^ and we showed that neuropeptide Y (NPY), which activates PKC in syncytiotrophoblast,^54^ also inhibited TauT activity in villous explants.^41^ The concentration of NPY in the serum of obese pregnant women has not been reported but the concentration is elevated in non-pregnant obese individuals,^55^ raising the possibility that NPY could downregulate placental TauT activity in maternal obesity.

Another post-translational modification of TauT that could downregulate activity in maternal obesity is the nitration of tyrosine residues on the transporter protein. Kulanthaivel *et al.*^56^ showed that tyrosine nitration of TauT significantly inhibited the activity of the transporter in syncytiotrophoblast microvillous plasma membrane. *In situ*, placental tyrosine groups are nitrated by peroxynitrite, generated in conditions of elevated nitrative stress^57^ and exposure of villous fragments to nitrative stress *in vitro* significantly reduced TauT activity.^58, 35^ Increased placental nitrative stress is evident in maternal obesity^23^ and could contribute to the reduction in placental TauT activity.

In summary, this study demonstrates that TauT activity, but not expression, is downregulated in placentas of obese women having otherwise normal pregnancies, a finding that is related to the severity of obesity. This reduction in TauT activity could lower taurine in syncytiotrophoblast, increase susceptibility to oxidative stress and inflammatory cytokines and reduce taurine delivery to the fetus, compromising fetal development. Understanding mechanisms that link raised maternal BMI to reduced TauT activity will allow development of interventions to restore uptake of taurine into the placenta to improve pregnancy outcome for obese mothers.

## Figures and Tables

**Table 1:** Demographics of the study participants

*Characteristics*	*Ideal weight (BMI 18.5*–*24.9)* n*=22*	*Overweight (BMI 25*–*29.9)* n*=12*	*Obese class I (BMI 30*–*34.9)* n*=13*	*Obese class II (BMI 35*–*39.9)* n*=7*	*Obese class III (BMI ⩾40)* n*=7*
BMI range (kg m^−2^)	22 (19–24)	27 (25–29)	32 (30–34)	37 (36–37)	42 (40–49)
White British (*n*)	13 (59%)	8 (66%)	6 (46%)	5 (71%)	5 (71%)
Asian (*n*)	5	2	2	—	1
Black Caribbean (*n*)	—	1	2	—	—
African (*n*)	3	1	1	1	1
Middle Eastern (*n*)	—	—	2	—	—
Mixed race (*n*)	1	—	—	1	—
Maternal age (years)	30 (23–40)	31 (21–36)	31 (21–40)	32 (20–39)	31 (24–36)
Parity	1 (0–3)	1 (0–6)	1 (0–3)	1 (1–3)	2 (0–4)
Smoking (*n*)	—	—	3	1	1
Mode of delivery	Vaginal—8; CS—14 (63%)	Vaginal—4; CS—8 (66%)	Vaginal—4; CS—9 (69%)	Vaginal—0; CS—7 (100%)	Vaginal—2; CS—5 (71%)

Abbreviations: BMI, body mass index; CS, Caesarean section.

Data are median and range. (%)=% of total. Smoking=cigarettes per day.

**Table 2:** Clinical characteristics of the infants of the study participants

*Maternal BMI group*					
*Characteristics*	*Ideal weight (*n*=22)*	*Overweight (*n*=12)*	*Obese class I (*n*=13)*	*Obese class II (*n*=7)*	*Obese class III (*n*=7)*
Birth weight (g)	3380 (2450–4260)	3530 (3140–4360)	3640 (2960–4330)	3560 (2920–4100)	3400 (3175–4100)
Placental weight (g)	528 (349–680)	550 (440–830)	561 (460–910)	590 (485–766)	560 (446–785)
FW:PW	6.4 (5.2–9.4)	6.3 (5.0–8.6)	6.1 (4.4–7.9)	5.4 (4.6–6.9)	6.4 (4.9–8.5)
IBC	47 (5–86)	55 (8–89)	65 (23–88)	49 (12–83)	56 (19–89)
Gestational age (weeks)	39.5 (37.0–41.8)	39.5 (38.7–42.0)	39.2 (37.0–41.8)	39.1 (38.1–40.4)	39.0 (38.0–40.4)

*Sex*					
Male	9	6	6	3	3
Female	13	6	7	4	4

Abbreviations: BMI, body mass index; FW:PW, fetal/placental weight ratio; IBC, individualized birth weight centile.

Data are median and range. Placental weight=blotted weight minus cord and membranes.
